# The Role of Acidic Residues in the C Terminal Tail of the LHCSR3 Protein of *Chlamydomonas reinhardtii* in Non-Photochemical Quenching

**DOI:** 10.1021/acs.jpclett.1c01382

**Published:** 2021-07-19

**Authors:** Franco V. A. Camargo, Federico Perozeni, Gabriel de la Cruz Valbuena, Luca Zuliani, Samim Sardar, Giulio Cerullo, Cosimo D’Andrea, Matteo Ballottari

**Affiliations:** † IFN-CNR, Dipartimento di Fisica, Politecnico di Milano, Piazza L. da Vinci 32, 20133 Milano, Italy; ‡ Dipartimento di Biotecnologie, Università di Verona, Strada Le Grazie 15, 37134 Verona, Italy; § Istituto Italiano di Tecnologia, Center for Nano Science and Technology, via Pascoli 70/3, 20133 Milano, Italy

## Abstract

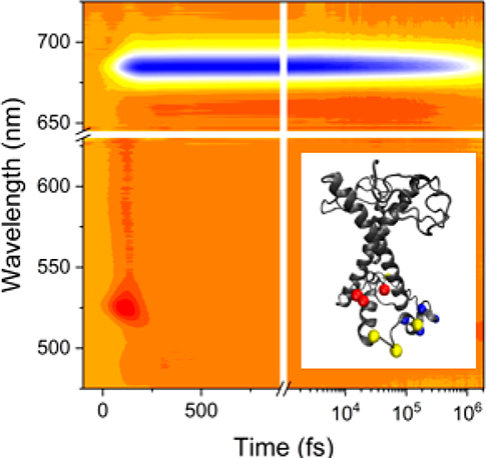

Light-harvesting complex stress-related (LHCSR) proteins in green algae are essential for photoprotection via a non-photochemical quenching (NPQ), playing the dual roles of pH sensing and dissipation of chlorophylls excited-state energy. pH sensing occurs via a protonation of acidic residues located mainly on its lumen-exposed C-terminus. Here, we combine in vivo and in vitro studies to ascertain the role in NPQ of these protonatable C-terminal residues in LHCSR3 from *Chlamydomonas reinhardtii*. In vivo studies show that four of the residues, D239, D240, E242, and D244, are not involved in NPQ. In vitro experiments on an LHCSR3 chimeric protein, obtained by a substitution of the C terminal with that of another LHC protein lacking acidic residues, show a reduction of NPQ compared to the wild type but preserve the quenching mechanism involving a charge transfer from carotenoids to chlorophylls. NPQ in LHCSR3 is thus a complex mechanism, composed of multiple contributions triggered by different acidic residues.

Photosynthetic organisms rely on the photochemical conversion of the absorbed light energy for their survival. However, an excessive illumination can be harmful for them, as it can lead to the formation of reactive oxygen species (ROS) that induce photodamage and, in extreme cases, even cell death.^1−4^ When the absorbed light power exceeds the capacity to regenerate nicotinamide adenine dinucleotide phosphate (NADP^+^) and adenosine diphosphate (ADP), precursors of NADPH and adensosine triphosphate (ATP), the photosynthetic apparatus undergoes an overexcitation. From this point, the production of increasing amounts of ROS can lead to cellular damage.^5^

A series of photoprotective mechanisms has been evolved to mitigate photodamage, whose onset depends on the exposure time to the incident light. The most rapidly activated mechanism is called non-photochemical quenching (NPQ) and leads to a dissipation of the absorbed light energy into heat by a nonradiative relaxation of the photoexcited chlorophylls (Chls), in parallel to the usual photochemical quenching pathway, which involves an excitation energy transfer (EET) to the reaction center.^5,6^ NPQ is a multicomponent phenomenon that consists of several processes occurring over a variety of time scales: energy-dependent feedback deexcitation quenching (qE),^7^ state transition-dependent quenching (qT),^8^ and a slowly relaxing quenching component (qI) that could be partially related to photodamage and chlorophyll degradation.^9^ Among NPQ mechanisms, qE has the fastest response, which is triggered by the acidification that occurs within the lumen of the thylakoids upon saturation of the photosynthetic apparatus caused by an intense illumination.

In *Chlamydomonasreinhardtii*, a model organism for green algae, two light-harvesting complexes (LHCs), the LHCSR1 and LHCSR3 proteins, have been reported to be essential for an qE induction.^10−12^ These LHC subunits are differently expressed in *C. reinhardtii*: both are upregulated in high light conditions, while a high CO_2_ availability downregulates the LHCSR3 expression and triggers LHCSR1.^13,14^ Both proteins are located inside the thylakoid membrane and possess protonatable residues exposed to the luminal side that are able to sense a pH acidification and trigger a heat dissipation.^10,15^ LHCSRs thus perform both the sensing and quenching roles in the qE process. Between LHCSR1 and LHCSR3 the latter has been reported to have the main role in a qE induction.^12,16^

The detailed molecular mechanisms of qE are still under debate, and several nonmutually exclusive models have been proposed. Mainly, these mechanisms involve the interaction of Chls with carotenoids (Cars), either through an EET from the Chl singlet excited state to dark Car excited states,^17−20^ or through a charge transfer (CT) from Cars to Chls, forming a Car radical cation in the process.^21−24^ Other studies pointed out the effect of Chl-Chl interactions in the presence of protein aggregation.^25,26^ Recently we reported the identification of multiple quenching mechanisms in the in vitro refolded LHCSR3 using a combination of picosecond time-resolved photoluminescence (TRPL) and femtosecond transient absorption (TA) spectroscopy. We observed a pH-triggered electron transfer from Chl to Car, which was however unable to fully account for the NPQ process, suggesting the presence of an aggregation-dependent quenching due to protein–protein interactions.^27^

pH sensing in LHCSR3 occurs via a protonation of acidic residues (aspartic and glutamic acids) located on the lumen-exposed side of the protein, mostly concentrated in the C-terminal portion, or in between the α-helices.^10,15^ Unlike most of the other LHCSR subunits from different microalgae species, the LHCSR C-terminal of *C. reinhardtii* is especially rich in protonatable residues, hinting at a probable pH-sensing role of this protein fragment.^10,15^ The importance of protonation as a trigger for quenching is well-known, and previous works showed the involvement of specific residues in the activation of NPQ.^10,15,28^ Still, the detailed role of these protonatable residues in triggering the LHCSR3 quenching mechanisms is not understood.

Here we combine in vivo and in vitro studies to ascertain the role of the protonatable C-terminal residues of LHCSR3 from *C. reinhardtii* in the NPQ process. In vivo experiments are performed on the *npq4lhcsr1* mutant, which is depleted of all LHCSR proteins, complemented with a mutant of LHCSR3 lacking four protonatable residues. In vitro experiments are performed with a chimeric fusion protein in which the C terminus of LHCSR3 is substituted with that of another LHC protein, LHCBM6, which completely lacks protonatable residues. Our results indicate that protonatable residues at the C-terminal loop of LHCSR3 are specifically involved in the pH-dependent activation of an LHCSR3 quenching mechanism, which is however not based on Chl to Car CT.

LHCSR3 from *C. reinhardtii* presents a peculiar C-terminal containing 10 acidic residues (the four aspartates D239, D240, D244, and D254 and the six glutamates E221, E224, E231, E232, E237, and E242), as shown in Figure S1 in the Supporting Information. A phylogenetic analysis of LHCSR-like protein sequences, reported in Figure S2, shows that sequences with a greater than 50% conservation of protonatable residues can be found in evolutionarily related species. Among the different acidic residues, the highest residue conservation was found in the case of E221 and E224, which indeed have been already reported to have a role in LHCSR3 pH sensing.^10^ In the case of the other protonatable amino acids, a cluster of four residues, D239, D240, E242, and D244, was characterized by a high solvent accessibility but a low conservation among the different LHCSR-like subunits, which can indicate a possible role in quenching mechanisms peculiar to *C. reinhardtii* (Figure S3).

To investigate the potential role of D239-D240-E242-D244 in LHCSR3 qE activity, we generated a site-specific mutant of the *lhcsr3.2* gene,^29^ which was used to complement the *npq4 lhcsr1* mutant. The wild-type (WT) *lhcsr3.2* gene was also used for *npq4lhcsr1* complementation as a control. The LHCSR3 accumulation per Photosystem II (PSII) was then evaluated by a western blot.^29^ Both for WT and D239N-D240N-E242Q-D244N (hereafter named the Q mutant, for quadruple) LHCSR3 variants, complemented lines with different levels of protein accumulation were obtained upon a high light adaptation, likely due to a position effect. In fact, the point in which the exogenous sequence is integrated in the host genome is random and can positively or negatively affect the gene expression capabilities. The NPQ induction of the complemented lines was then measured by a pulse amplitude modulation fluorescence in whole cells (Figure 1b), obtaining as a result an NPQ effect proportional to the LHCSR3 protein content.^12,30^ Indeed, by plotting the maximum of the NPQ (Figure 1c) and of the qE (Figure 1d) component as a function of the ratio between LHCSR and PSII,^29^ a linear correlation is observed for both WT and Q lines. These results demonstrate that the substitution of the acidic residues D239-D240-E242-D244 with nonprotonatable residues does not lead to a measurable perturbation of the overall pH sensing and NPQ activity of LHCSR3.

**Figure 1:**
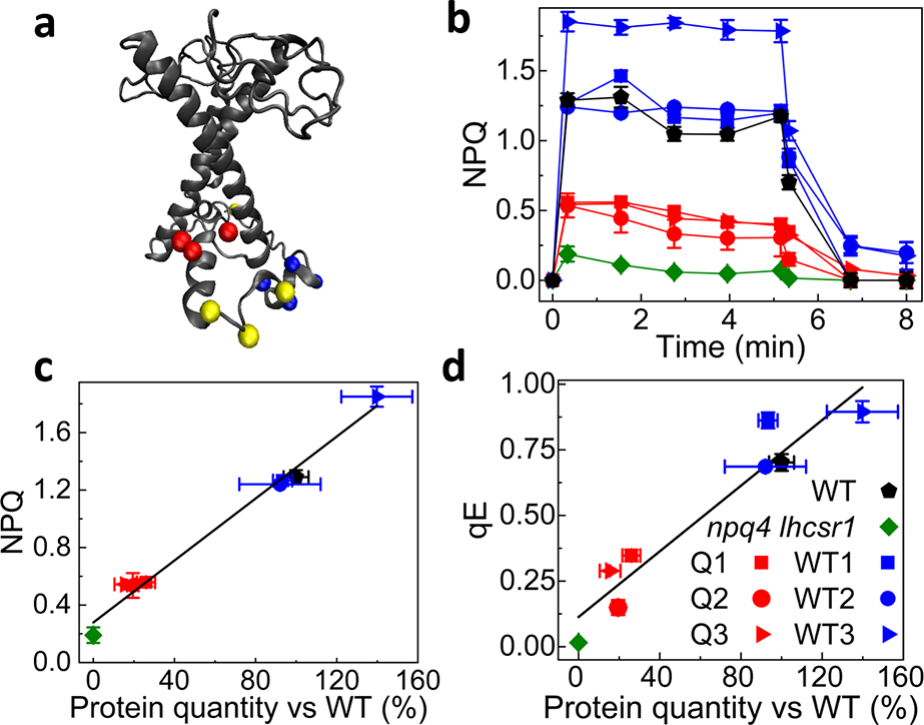
(a) Structural model of LHCSR3 obtained by sequence alignment with CP29 (PDB No. 3PL9); protonatable residues are highlighted as yellow, red, and blue beads. Red beads (D117, E221, E224) are the protonatable residues described elsewhere;^10^ blue beads are the protonatable residues (D239-D240-E242-D244) substituted in the Q mutant, and yellow beads (E231, E233, E237 and D254) are the remaining acidic residues. (b) NPQ traces for WT, *npq4 lhcsr1* complemented with LHCSR3 WT (WT1, WT2, WT3) or with the Q mutant (Q1, Q2, Q3). Light was turned on from 0 to 5 min and then turned off. (c) Linear regression of NPQ vs LHCSR3 content per PSII. (d) Linear regression of qE vs LHCSR3 content per PSII. (b–d) Error bars are reported as standard deviations (*n* = 3).

In vivo results suggest either the absence of a specific role for these four residues or the presence of a redundant mechanism in which the lack of specific protonatable sites can be compensated by the others. These results motivated us to generate a new LHCSR3 mutant in which eight acidic residues at the C-terminus were substituted by neutral ones. In addition to the above-mentioned D239N, D240N, E242Q, and D244N mutations, other mutations were introduced in the same coding sequence inducing E231Q, E233Q, E237Q, and D254N substitution. This new mutant sequence, carrying eight mutations at the C-terminus, was again used to complement the *npq4lhcsr1* mutant and test, after a high light exposure of colonies, NPQ, and LHCSR3 protein accumulation. One hundred and ninety-two resistant colonies were screened by immunoblotting after a high light exposure, but none of them was found to accumulate a detectable amount of protein. We conclude that the mutations introduced on the eight acidic residues at the C-terminus destabilize the LHCSR3 protein, preventing its accumulation in vivo.

For this reason, a new LHCSR3 mutant was generated by a substitution of the C-terminus of LHCSR3 with the C-terminus of another LHC protein found in the same host organism, the LHCBM6 subunit of *C. reinhardtii* (Figure S4). We chose LHCBM6 as the candidate, considering that it does not contain acidic residues in the C-terminal region.^31^ This chimeric version of LHCSR3 (called hereafter LHCSR3-BM6) was used to complement the *npq4lhcsr1* mutant, but once again, among the 288 screened colonies, none was found to accumulate the mutated protein.

The inability to characterize in vivo the mutant lacking eight protonatable residues at the C-terminus motivated us to proceed with an in vitro characterization. LHCSR3 WT and LHCSR3-BM6 were thus overexpressed in *Escherichia coli*, purified, and refolded in vitro, as previously described.^32^ It is worth mentioning that, in the LHCSR3-BM6 chimeric protein, the acidic residues D119, E221, and E224, previously reported to be involved in the LHCSR3 pH-sensing activity in vivo, are still present,^10^ while the eight acidic residues at the C-terminus, proposed to act as a pH-sensing switch in LHCSR3, are absent.^15^

In a steady state, both the LHCSR3 WT and the LHCSR3-BM6 mutant showed an absorption peak at 679.2 nm and a photoluminescence (PL) peak at 683.5 nm, in agreement with previous findings (Figure S5).^27,32^ Pigment binding properties of both refolded proteins, reported in Table S1 in the Supporting Information, show no significant differences between the WT and the mutant. This, along with the overall similarity of absorption and PL spectra, suggests that no dramatic changes occur in the overall structure and pigment binding properties of LHCSR3 upon substitution of the C-terminal region with that of LHCBM6. It is worth noting that these conclusions are based on results obtained with recombinant proteins refolded in vitro, thus not including possible effects of the thylakoid environment.

To investigate the possible effects on the quenching activity of LHCSR3 induced by the substitution of its C-terminus, we compared the transient optical response of WT LHCSR3 and LHCSR3-BM6 after a selective excitation of Chls, using both TRPL and TA spectroscopies. The combination of these techniques is particularly powerful, since TRPL measures the lifetime of the emitting excited states, such as the Chl singlet excited state (^1^Chl*), while TA also allows the identification of nonemitting states, such as the Chl and Car triplet states (^3^Chl*, ^3^Car*) and the Car dark state S_1_ as well as Car radical cations. It is well-known that an aggregation has an important effect on the fluorescence lifetime in LHCSR.^10,27^ Indeed, proteins in a low detergent concentration undergo clustering that favors protein–protein interactions and a potential nonlinear annihilation of the excitations. Here, to avoid effects ascribable to small detergent differences, all measurements were performed maintaining a detergent concentration of 0.03% of α-dodecyl maltoside. The aggregation states of refolded LHCSR3 proteins at pH 7.5 and 5 were monitored by the fluorescence emission at 77 K, where the possible formation of aggregates is detectable by the appearance of new red-shifted fluorescence bands above 700 nm.^33^ That was not the case for any of our samples, as reported in Figure S6.

Figure 2a displays the TRPL maps of LHCSR3-BM6 at pH 5, while Figure 2c compares the normalized TRPL kinetics, integrated over the 650–750 nm range, for LHCSR3 WT and LHCSR3-BM6 at pH 7.5 and 5, respectively. TRPL maps, PL spectra integrated over the full experimental time window, and the results of the biexponential fits are shown in Figures S7 and S8 and in Table S2 in the Supporting Information, respectively. LHCSR3 WT at pH 5 shows a faster decay than at pH 7.5, in agreement with previous studies that revealed a pH-dependent quenching mechanism in the same protein^27^ or in a related one, LHCSR1.^34^ Interestingly, the same pH dependence is also present in the LHCSR3-BM6 samples but with a clearly reduced quenching with respect to the WT. Hence, we conclude that the pH-induced quenching is reduced, but not completely suppressed, in the LHCSR3-BM6 mutant. When a TRPL analysis was performed to compare LHCSR3 WT and LHCSR3-Q, the fluorescence kinetics were similar between the WT and mutant (Figure S9), consistently with the similar LHCSR3 quenching activity evinced by an in vivo analysis (Figure 1).

**Figure 2:**
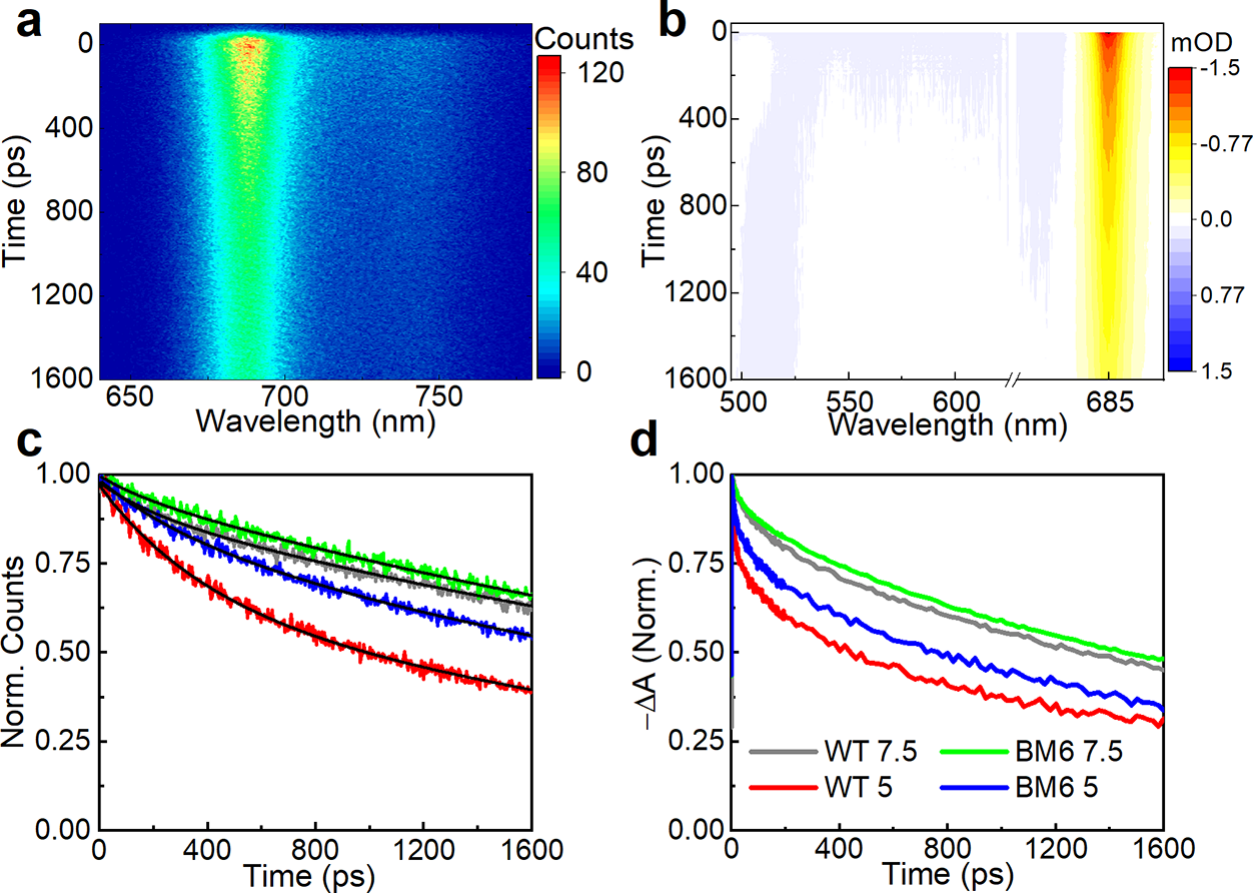
(a) TRPL and (b) TA maps of the LHCSR3-BM6 mutant at pH 5. (c) TRPL time traces for WT and mutant samples at pH 5 and 7.5. The TRPL data were integrated between 650 and 750 nm, and the black lines are multiexponential fits to the data. (d) TA kinetic traces at 685 nm following a 630 nm photoexcitation.

To shed further light on the mechanisms presiding over quenching in LHCSR3-BM6, we performed TA spectroscopy. Figure 2b shows the TA maps of LHCSR3-BM6 at pH 5 after a selective Chl excitation at 630 nm, while Figure 2d compares pH-dependent normalized TA kinetics of WT and mutant samples at 680 nm. TA maps and TA spectra at selected time delays are reported in Figures S10 and S11, respectively. To avoid kinetic distortions due to singlet–singlet annihilation,^35^ we performed fluence-dependent measurements (see Figure S12 in the Supporting Information) and selected fluences for which annihilation effects are negligible (7 μJ/cm^2^ for the pH 7.5 WT sample and 2 μJ/cm^2^ for the other samples). TA spectra at early times (see Figure S11) show the same features for all samples: a strong negative peak around 680 nm due to ^1^Chl* ground-state bleaching (GSB) and stimulated emission (SE) and a broad positive band extending from 450 to 600 nm corresponding to a ^1^Chl* photoinduced absorption (PA). Chl GSB/SE kinetics (Figure 2d) agree with TRPL decays (Figure 2c), showing a faster GSB decay at a lower pH in LHCSR3 WT and LHCSR3-BM6, with the latter exhibiting a reduced quenching. The TRPL and TA data thus consistently indicate that LHCSR3-BM6, due to the absence of eight protonatable residues in the C-terminal region, displays a reduced pH-dependent qE activity.

Since most proposed mechanisms for qE are associated with Chl-Car interactions, we then focused on the spectral region of Car activity (450–600 nm), which overlaps with the broad ^1^Chl* PA. Since the 630 nm pump pulses do not directly excite the Cars, any observed signal from the Cars must be assigned to the interaction of ^1^Chl* with them. Figure S11 reports TA spectra in the 450–600 nm wavelength range at different delays. The broad positive band is assigned to PA from ^1^Chl*. On top of this band, we observe the formation of a narrower negative band peaking at ∼490 nm over tens of picoseconds. We assign the signal to Car GSB, in agreement with previous results.^34,36,37^Figure 3a compares the normalized TA time traces of all samples at 500 nm in the first 250 ps. We observe a positive signal that partially decays on the tens of picoseconds time scale. This decay, which is assigned to the formation of the Car GSB overlapping with the ^1^Chl* PA, is more pronounced for pH 5 than for pH 7.5. This result confirms the involvement of Cars in the pH-dependent qE process in LHCSR3. Nevertheless, the similarities between the kinetics of the WT sample and the LHCSR3-BM6 mutant at the same pH values are remarkable.

**Figure 3:**
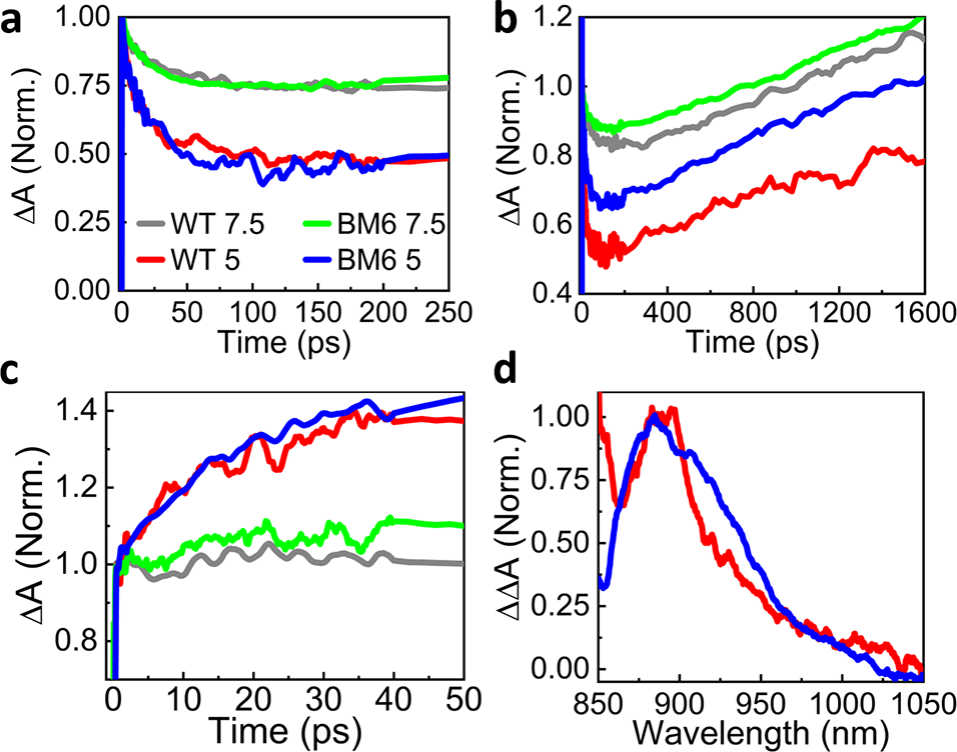
LHCSR3 WT and LHCSR-BM6 TA kinetics after a 630 nm excitation of (a) Car GSB at 500 nm, (b) Car triplet formation at 510 nm, and (c) Car radical cation formation at 880 nm. (d) Double difference ΔΔA spectra of the pH 5 sample showing a lutein radical cation formation at ∼880 nm.

On the nanosecond time scale, we also see a rising PA signal around 510 nm (Figure 3b), which can be attributed to the buildup of the Car triplet state, ^3^Car*.^38^ This state is formed by a triplet–triplet energy transfer from ^3^Chl* to ^3^Car*, following an intersystem crossing (ISC) in Chl, according to the scheme ^1^Chl*→ ^3^Chl*→ ^3^Car*. We observe that the Car triplet formation is more pronounced at pH 7.5 as opposed to pH 5 and in the mutant with respect to WT. This is consistent with a longer ^1^Chl* lifetime due to a reduced qE, which makes the ISC process more likely. These results show a correlation between the ^1^Chl* lifetime quenching observed in TRPL and TA experiments and the buildup of the Car GSB.

A possible ^1^Chl* quenching mechanism is the EET from the ^1^Chl* state to a Car dark state, such as S_1_ or the recently proposed S*.^17−19^ The population of a Car dark state can be measured indirectly by observing the S_1_ → S_n_ PA, which, depending on the Car involved, peaks in the 530–580 nm range.^34^ Because of the short Car S_1_ lifetime, this transition can only be detected when the state is highly populated. The TA dynamics at 570 nm (Figure S13) do not show a formation, thus indicating that the qE mechanism involving EET to a Car dark state is either inactive or of minor importance.

An alternative ^1^Chl* quenching mechanism is the CT from the Cars, which results in a Car radical cation formation. To probe this process, we extended our TA detection window to the near-infrared (NIR) region (850–1050 nm), where Car radical cations absorb.^21^ TA spectra in the NIR at different time delays are shown in Figure S14. At early times (500 fs) we observe a broad positive band, which we assign to ^1^Chl* PA.^21^ On the 20 ps time scale this band remains substantially constant for the pH 7.5 samples, while it displays a clear buildup at pH 5 for both the WT and the mutant samples. The TA dynamics at 880 nm, reported in Figure 3c, show a signal growth by ∼40% for the pH 5 samples.

We assign the NIR TA buildup in the pH5 samples to the formation of a Car radical cation. To determine which Car is involved in the process, we subtract the TA spectra at 500 fs from those at 20 ps, generating double difference spectra ΔΔA. Normalized ΔΔA spectra at pH 5 are shown in Figure 3d for both WT and mutant samples. The peaks at ∼880 nm are in good agreement with the PA spectrum of the lutein radical cation.^27,39^ Taken together, the visible and NIR TA data indicate the quenching of ^1^Chl* via a CT from the CAR, which results in the simultaneous buildup of the CAR GSB (Figure 3a) and of the PA of the CAR radical cation (Figure 3c). Notably, the data in Figure 3a,c do not highlight significant differences between the WT and the LHCSR3-BM6 mutant, demonstrating that the qE mechanism related to CT from the Cars is not triggered by the protonatable residues on the C-terminus.

In conclusion, our work sheds new light on the molecular mechanisms underlying the pH-dependent quenching activity of LHCSR3. First, using in vivo measurements, we found that four of the acidic residues at the C-terminus previously reported to be involved in pH sensing, namely, D239, D240, E242, and D244, are not essential, since the LHCSR3 quenching activity is similar when they are substituted with neutral ones. The pH-sensing property of LHCSR3 is thus likely based on a redundant role of the different acidic residues. When the whole C-terminus of LHCSR3 was substituted with the C-terminus of an LHC protein with no pH-sensing properties, in vitro experiments showed that the pH-dependent protein activation as a quencher was reduced, consistently with previous results,^15,28^ but not fully impaired. In particular, the quenching mechanism based on the Chl-Car interaction and a lutein radical cation formation was found to be unaltered even in the LHCSR3-BM6 mutant and could be likely attributed to the pH sensing of the remaining protonatable residues E221, E224, and D117, whose role in the LHCSR3 quenching activity has been demonstrated by in vivo mutagenesis and complementation.^10^ The possible involvement of E221, E224, and D117 protonatable residues on the carotenoid-chlorophyll charge transfer quenching needs to be proven by future work on recombinant LHCSR3 protein variants.

A pH-dependent activation of LHCSR3 quenching mechanisms is thus composed of multiple contributions: protonatable residues E221, E224, and D117 are involved in a pH-dependent activation of qE via a lutein radical cation formation,^10^ while acidic residues at the C-terminal loop, E231, E233, E237, D239, D240, E242, D244, and D254, are involved in the pH-dependent activation of other LHCSR3 quenching mechanisms, which do not involve interactions between Chls and Cars, and can be putatively assigned to Chl-Chl interactions.

## Supporting Information Available

Materials and methods: sample preparation, time-resolved photoluminescence, transient absorption, global analysis, and supplementary data (PDF)
