# Supplementary material for: The Role of Acidic Residues in the C Terminal Tail of the LHCSR3 Protein of Chlamydomonas reinhardtii in Non-Photochemical Quenching
Source: J Phys Chem Lett. 2021 Jul 19;12(29):6895–900. doi: 10.1021/acs.jpclett.1c01382 (PMC8327309; doi:10.1021/acs.jpclett.1c01382)
Supplement: Supplementary file 1 — jz1c01382_si_001.pdf [file jz1c01382_si_001.pdf]

The Role of Acidic Residues in the C Terminal Tail of the LHCSR3 Protein of *Chlamydomonas reinhardtii* in Non-Photochemical Quenching

Franco V. A. Camargo[1,‡], Federico Perozeni[2,‡], Gabriel de la Cruz Valbuena[1,3], Luca Zuliani[2], Samim Sardar[3], Giulio Cerullo[1]*, Cosimo D'Andrea[1,3]*, Matteo Ballottari[2]*

[1] IFN-CNR, Department of Physics, Politecnico di Milano, P.za L. da Vinci 32, 20133 Milano, Italy

[2] Dipartimento di Biotecnologie, Università di Verona, Strada Le Grazie 15, 37134 Verona, Italy

[3] Istituto Italiano di Tecnologia, Center for Nano Science and Technology, via Pascoli 70/3, 20133 Milano, Italy.

Supporting information

MATERIALS AND METHODS

Structural model and bioinformatics analysis

A model of the LHCSR3 structure was obtained as described in reference [1] by using the structure of CP29 (PDB 3PL9) as a reference.[2] The protein structure was then visualized by Chimera 1.13 software. Solvent accessibility for C-terminal region (residues 220-259) of *C. reinhardtii* LHCSR3 (XP_001696064.1) was predicted using I-TASSER software (Roy et al 2010).[3] All alignments and phylogenetic tree were done using Clustal Omega, BLAST or NCBI tree viewer software with parameters listed in each figure.

LHCSR3 *in vitro* reconstitution and sample preparation

LHCSR3 in vitro refolding was obtained as previously reported in Bonente et al 2011.[4] In particular, lhcsr3 cDNA was cloned in pET28-His vector for overexpression in *E.coli* removing the coding sequence for the first 14 residues, being the predicted transit peptide for chloroplast localization.[4,5] For LHCSR3-Q mutant the aspartate and glutamate residues D239-D240-E242-D244 were substituted respectively by asparagine or glutamine by using QuikChange® Site-Directed Mutagenesis Kit according to the manufacturer's instructions (Ballottari et al 2016). For LHCSR3-BM6 mutant the C-term region of LHCSR3 was substituted by PCR with the 60bp region of LHCSBM6. LHCSR3 apoproteins were purified from *E. coli* as reported by Liguori et al.[6] and refolded *in vitro* in presence of pigments as previously described.[1]

Samples, both for time resolved fluorescence and transient absorption, were prepared as reported in de la Cruz Valbuena, 2019.[7]

Steady state absorption and fluorescence

Absorption measurements were performed with a Cary 4000, Varian spectrophotometer. Steady state fluorescence measurements were performed with a BeamBio custom device equipped with an USB2000+ Ocean Optics spectrometer and custom LED light sources for excitation.

Pigment analysis

Pigment analysis were performed by HPLC as described in by Lagarde, Beuf, and Vermaas.[8] HPLC analysis was performed with a Jasco LC-4000 Extrema HPLC system (Jasco, Italy) equipped with a C18 column (Synergi 4u Hydro-RP 80A, Phenomenex, USA) and 350–750 nm diode array detector.

***In vivo* complementation of *npq4 lhcsr1 C. reinhardtii* mutant**

In vivo complementation of *npq4 lhcsr1* mutant was performed as described in Perozeni et al 2019.[5] The LHCSR3.2 gene (Cre08.g367400) was amplified from *C. reinhardtii* genomic DNA as previously described.[1] In particular, the DNA region amplified was from –1000bp upstream the 5' untranslated region (UTR) to 300bp downstream the 3' UTR.[5] Amplified DNA was then inserted in pBC1 vector as described by Ballottari et al.[1] Site-specific mutagenesis was performed on identified protonatable residues using QuikChange® Site-Directed Mutagenesis Kit according to the manufacturer's instructions.[1] Cells transformation and transformant selection was performed as described in Refs. [1,5]. Paromomycin was used as selective marker. Resistant colonies were then transferred in TAP medium at 25 °C in flasks with white light (70 μE m^{-2} s^{-1}, 16h light/8h dark photoperiod). High light acclimation was induced in WT (4A+ strain), *npq4 lhcsr1* mutant and transformant lines by growing cells in HS medium at 400 μE m^{-2} s^{-1}.[1] LHCSR3 protein content in the WT and complemented lines was analyzed by immunoblotting by using a specific antibody α-LHCSR3 (AS14 2766, Agrisera, Sweden). In order to retrieve the LHCSR3 content per PSII, the accumulation of PSII core subunits CP43 was monitored as a proxy of PSII by immunoblotting by using α-CP43 antibody (AS11 1787). [1,5]

NPQ analysis

NPQ induction curves were measured on *C. reinhardtii strains* by using a Dual-PAM 100 instrument (WALZ, Germany). NPQ analyses were performed on *C. reinhardtii* cultures in exponential phase acclimated to high light for 4 days. Measuring, saturating and actinic light was respectively 7, 5000 and 1200 µmol m^{-2} s^{-1}.

Time-Resolved photoluminescence

Time-resolved photoluminescence measurements were carried out using a Ti:sapphire laser (Chameleon Ultra II, Coherent) with a repetition rate of 80 MHz and pulse width of about 100 fs at FWHM. The laser output was set at 860 nm and then frequency doubled by a β-barium borate (BBO) crystal to generate the 430 nm excitation. The detection system consists in a spectrometer coupled with a streak camera (C5680, Hamamatsu) which provides spectral-temporal matrices with spectral and temporal resolutions of ~1 nm and ~20 ps, respectively. TRPL and TA measurements were carried out in cuvettes with optical path-length of 3 and 1 mm, respectively. The sample temperature has been maintained at 10 degrees Celsius to avoid protein unfolding.

Transient absorption

Transient absorption measurements were carried out using a home-made setup, based on a regeneratively amplified Ti:Sapphire laser (Libra, Coherent) at 800 nm wavelength with 100 fs pulse duration and 1 kHz repetition rate. A fraction of the fundamental wavelength was used to seed a non-collinear optical parametric amplifier (NOPA) delivering ~80 fs pulses at a central wavelength of 630 nm. The pump fluence was adjusted to minimize bimolecular recombination. The broadband probe beam was obtained through white light continuum generation by focusing the fundamental pulses either in a 2-mm-thick sapphire plate or in a 4-mm-thick YAG crystal, in combination with appropriate filters to reject the remaining light at 800 nm. The spectrum generated covered respectively the visible (470-700 nm) or the infrared (850-1050 nm) range. After the sample, the transmitted probe was sent to a spectrometer (SP2150 Acton, Princeton Instruments) and detected using a linear image sensor driven and read out by a custom-built board (Stresing Entwicklungsburo). For each probe wavelength, the differential absorption (ΔA) was measured as a function of the pump-probe delay. All measurements were acquired at magic angle (54.7°) polarization. Before each measurement, the beam profile of pump and probe beams was imaged at the overlap position using a camera, and the cross-sections obtained were used to calculate the fluence. The sample was kept at 12°C during all TA experiments. The signal levels of different TA scans were compared to check for possible sample degradation during each measurement, and that was never the case under these measurement conditions.

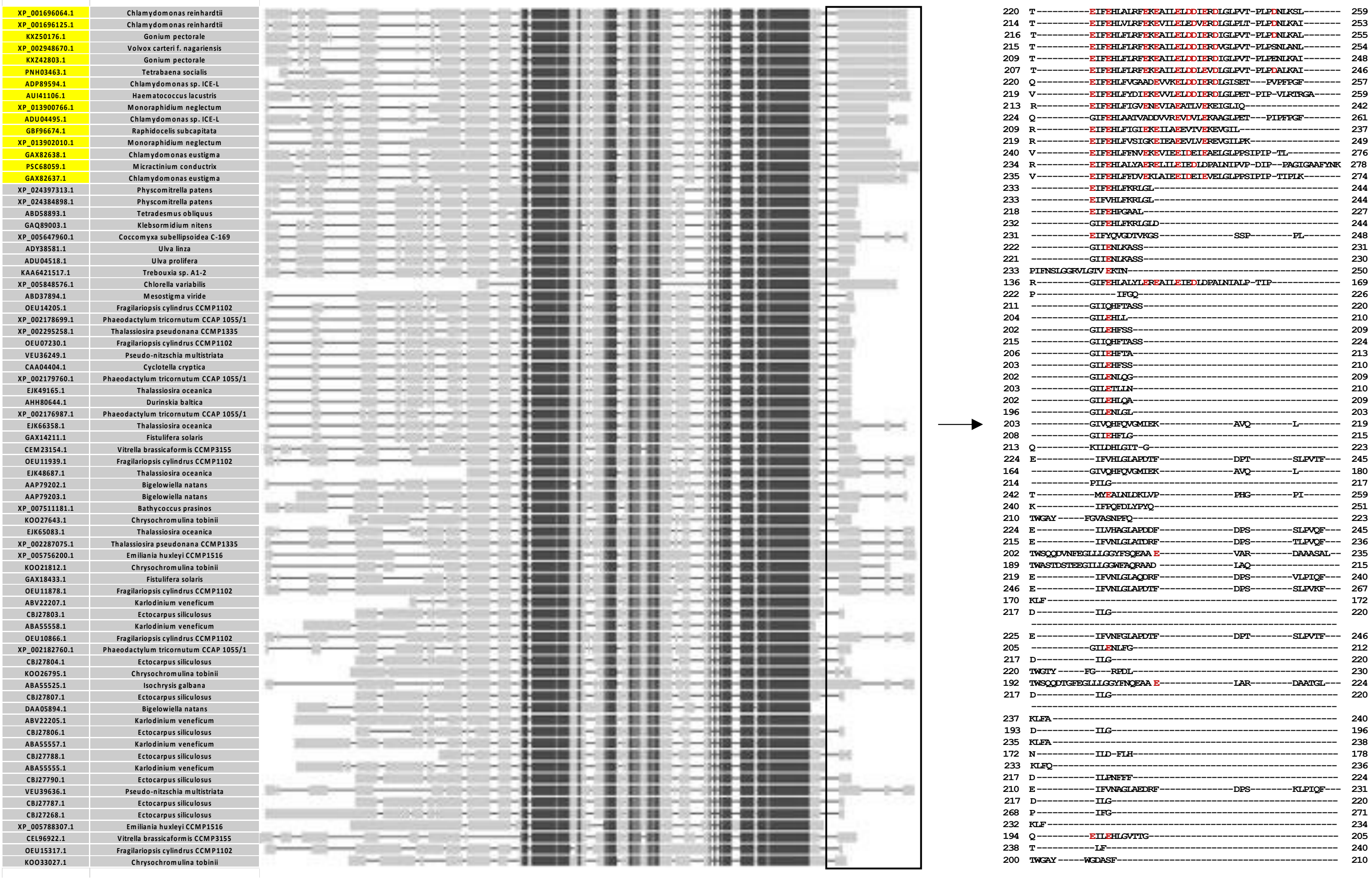

Figure S1. Alignment of LHCSR-like protein sequences. BLAST of LHCSR-like proteins using LHCSR3 from *Chlamydomonas reinhardtii* (XP_001696064.1). Sequences with e-value and score higher than $3e^{-39}$ and 144 respectively are listed with accession number and organisms and a graphic overview is presented. On right, focus on C-terminal region; conserved protonatable residues are written in red. Accession numbers highlighted in yellow represent proteins with protonatable residues conservation higher than 50%

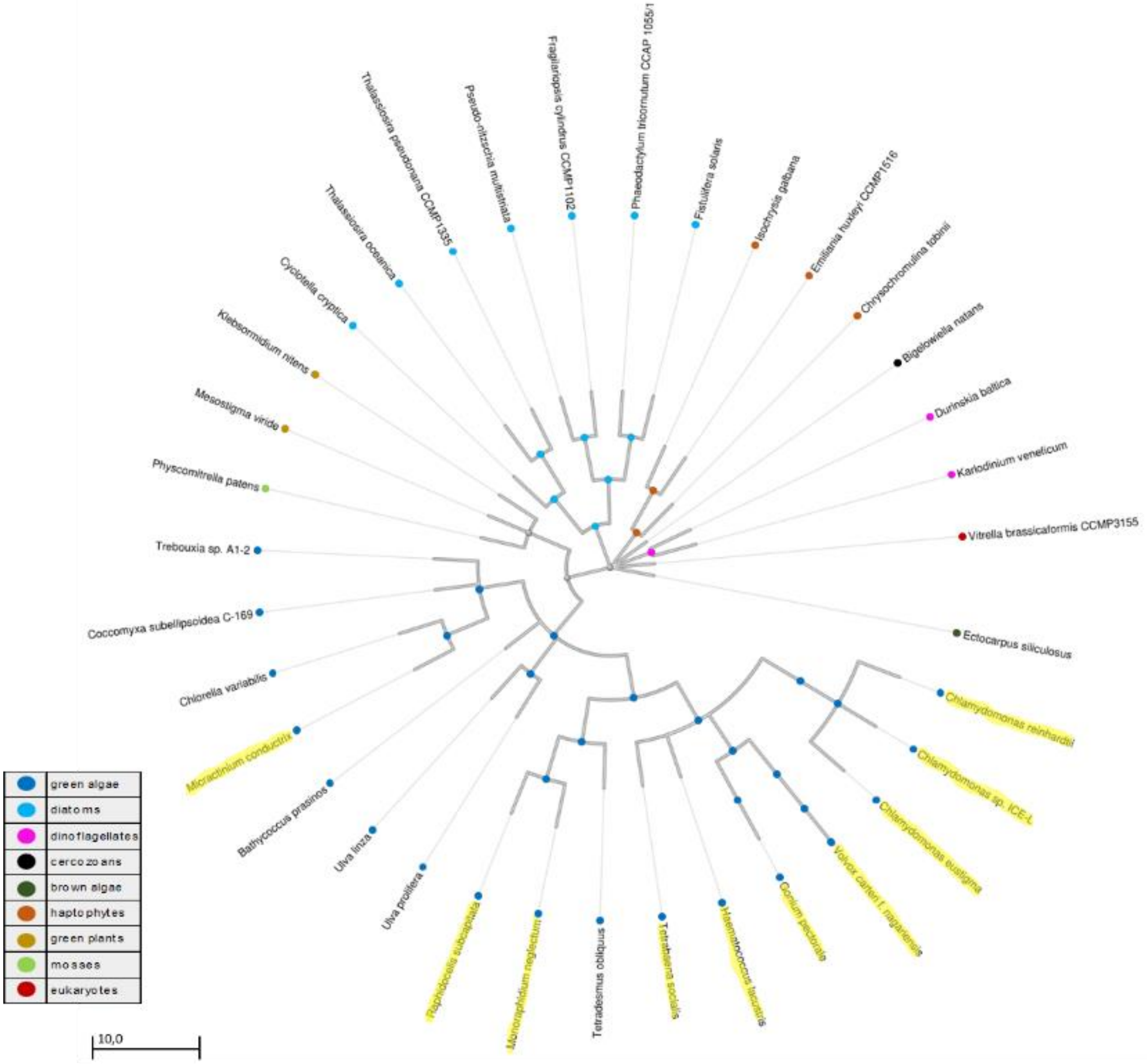

Figure S2. Phylogenetic tree of the organisms possessing LHCSR-like proteins. Organism listed in Figure S1 were subjected to a phylogenetic tree building using NCBI taxonomy database. Yellow highlighted organisms are those presenting protein with protonatable residues conservation higher than 50%, which are the same highlighted in Figure S1.

		E221			E224							E231		E233				E237		D239
Sequence	T	E	I	F	E	H	L	A	L	R	F	E	K	E	A	I	L	E	L	D
Solvent accessibility	4	2	1	0	3	1	0	2	4	1	0	4	4	1	1	1	2	2	0	3
Protonatable residues solvent accessibility		2			3							4		1				2		3
Residue conservation (all sequences)		3			5							2		2				2		2
conservation >50%)		9			10							8		9				10		7
TOTAL SCORE		3			0							6		4				4		6

	D240		E242		D244										D254					
Sequence	D	I	E	R	D	L	G	L	P	V	T	P	L	P	D	N	L	K	S	L
Solvent accessibility	4	0	4	5	4	2	2	1	3	3	0	3	1	3	6	4	0	4	7	5
Protonatable residues solvent accessibility	4		4		4										6					
Residue conservation (all sequences)	1		2		1										1					
conservation >50%)	6		9		5										3					
TOTAL SCORE	7		6		8										10					

Figure S3. Solvent accessibility and residues conservation. Solvent accessibility for C-terminal region (residues 220-259) of *C. reinhardtii* LHCSR3 (XP_001696064.1) was predicted using I-TASSER software which attributes to each residue a 0-9 score indicating the probability for solvent exposure (high score indicates higher probability). Residues conservation between all the sequences used for alignment and between 15 sequences showing protonatable residues conservation higher than 50% (same highlighted in figure S1 and S2). Conservation is expressed by a 0-10 score with 10 meaning conservation in all sequences and 0 in none. Total score, calculated as [(3*ResiduesConservation)-SolventAccessibility] is expressed as 0-10 score; high score means higher probability to be peculiar sites in *C. reinhardtii* based on residues conservation and solvent accessibility.

```
LHCSR3_WT      MLANVVSRKASGLRQTPARATVAVKSVSGRRTTAAEPQTAAPVAAEDVFAYTKNLPGVTA    60
LHCSR3_BM6     MLANVVSRKASGLRQTPARATVAVKSVSGRRTTAAEPQTAAPVAAEDVFAYTKNLPGVTA    60
               ************************************************************

LHCSR3_WT      PFEGVFDPAGFLATASIKDVRRWRESEITHGRVAMLAALGFVVGEQLQDFPLFFNWDGRV    120
LHCSR3_BM6     PFEGVFDPAGFLATASIKDVRRWRESEITHGRVAMLAALGFVVGEQLQDFPLFFNWDGRV    120
               ************************************************************

LHCSR3_WT      SGPAIYHFQQIGQGFWEPLLIAIGVAESYRVAVGWATPTGTGFNSLKDDYEPGDLGFDPL    180
LHCSR3_BM6     SGPAIYHFQQIGQGFWEPLLIAIGVAESYRVAVGWATPTGTGFNSLKDDYEPGDLGFDPL    180
               ************************************************************

LHCSR3_WT      GLKPTDPEELKVMQTKELNNGRLAMIAIAAFVAQELVEQTEIFEHLALRFEKEAILELDD    240
LHCSR3_BM6     GLKPTDPEELKVMQTKELNNGRLAMIAIAAFVAQELVEQTEIFEHLALRFANPTVNNAFA    240
               **************************************************  :  : :  :

LHCSR3_WT      IERDLGLPVTPLPDNLKSL   259
LHCSR3_BM6     FATKFT----PSA------   249
               :  .:      *
```

Figure S4. Alignment of LHCSR3 and LHCSR3-BM6 protein sequences. LHCSR3 and LHCSR3 peptide sequences. “*” indicates perfect alignment; “:” indicates a site belonging to group exhibiting strong similarity while “.” indicates a site belonging to a group exhibiting weak similarity. Putative transit peptide and substitute LHCBM6 C-terminal region are orange and blue respectively highlighted. Protonatable residues, exposed to the lumen, according to Ballottari 2016 are red written.[1]

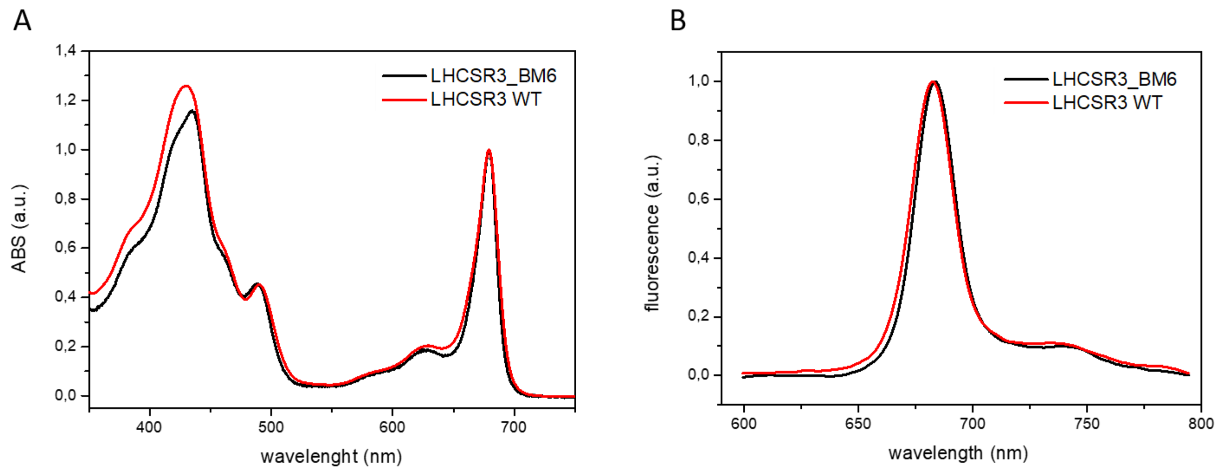

Figure S5. Absorption and fluorescence of LHCSR3 recombinant proteins. Absorption (A) and room temperature fluorescence (excitation at 440nm) (B) of LHCSR3 WT and LHCSR3-BM6.

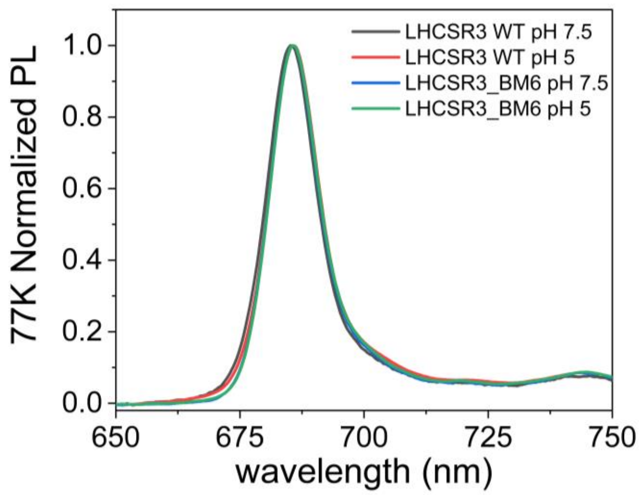

Figure S6. 77K fluorescence emission of LHCSR3 recombinant proteins. Fluorescence emission spectra at 77K (excitation at 440 nm) of LHCSR3 WT and LHCSR3-BM6.

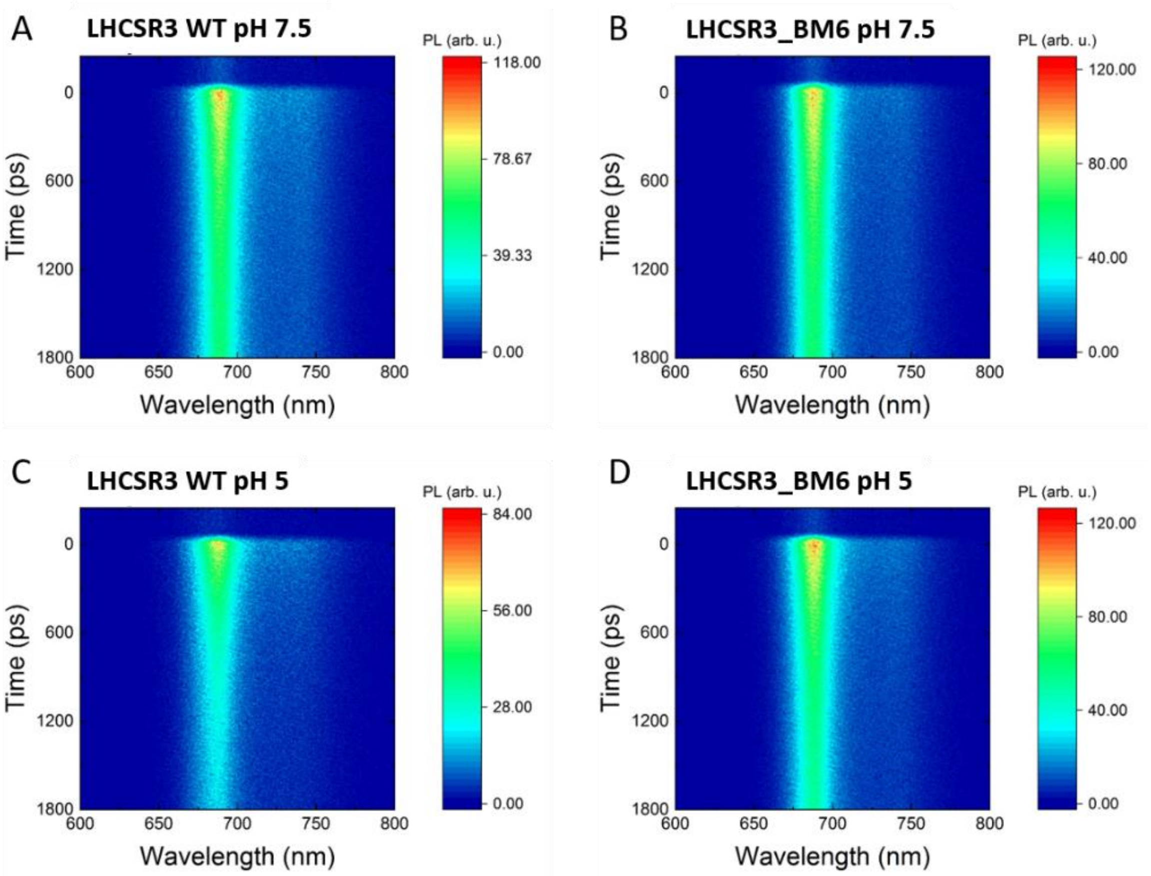

Figure S7. TRPL maps. TRPL maps (pump at 430nm) of LHCSR3 WT pH 7.5 (A) and pH 5 (C); LHCSR3-BM6 pH 7.5 (B) and 5 (D).

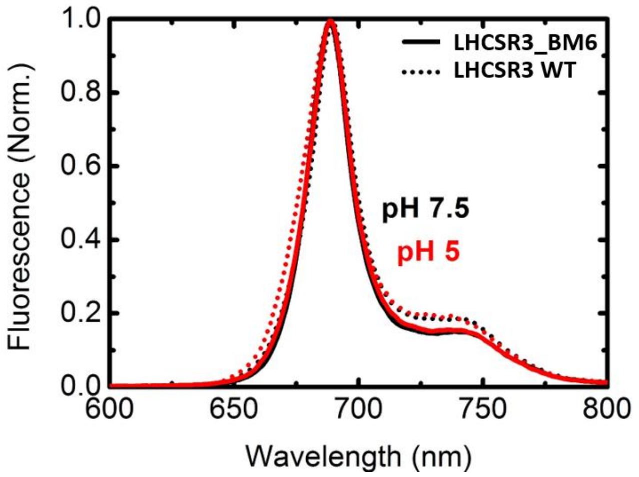

Figure S8. Time integrated TRPL spectra. Integrated TRPL spectra of LHCSR3 WT and LHCSR3-BM6 (pump at 430 nm, integration up to 1.8 ns).

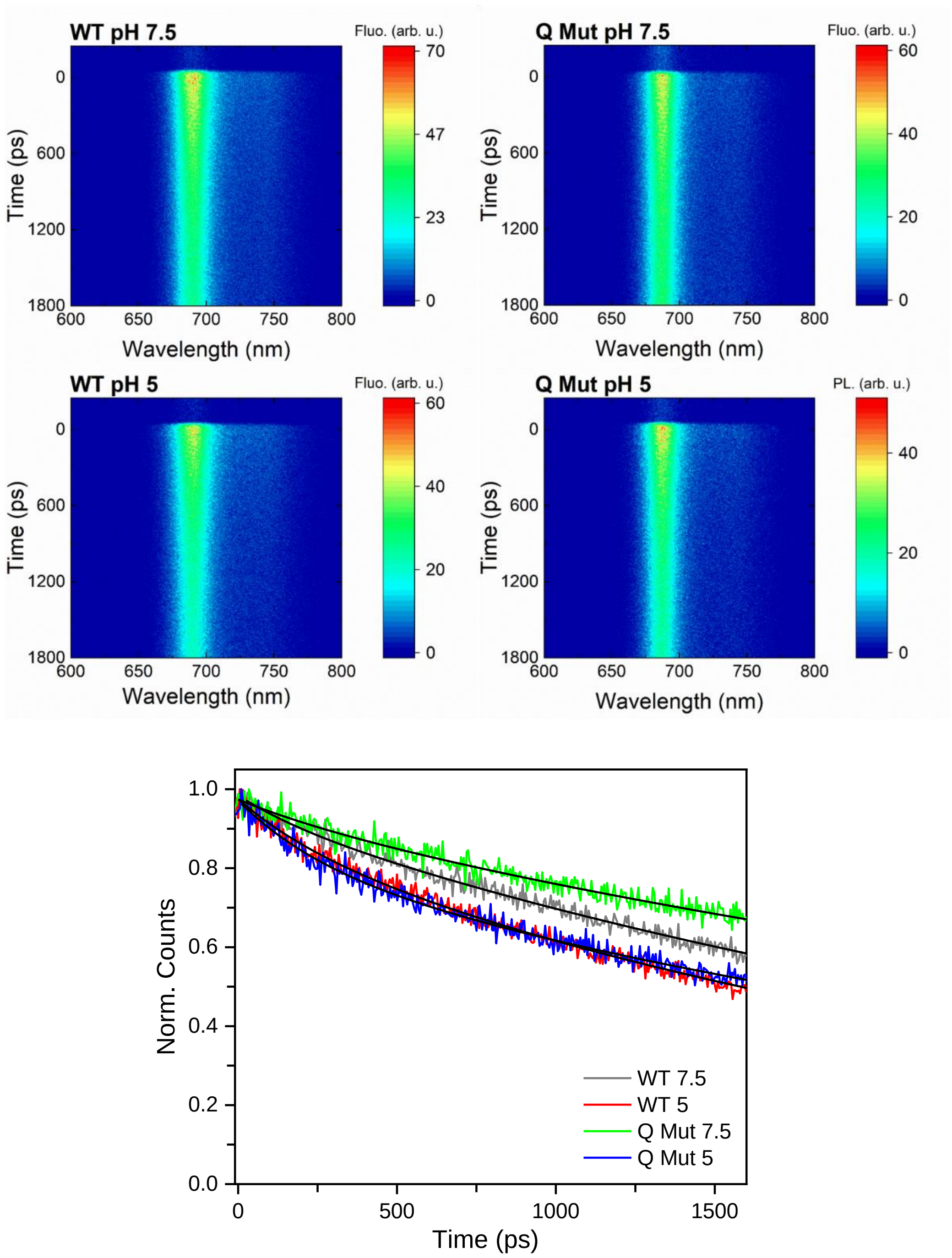

Figure S9. TRPL maps and time traces for LHCSR3 WT and LHCSR3-Q mutant at pH 5 and pH 7.5. The mutant and the WT, which acts as control sample, were prepared at the same time and with the same pigment mix. TRPL time traces for LHCSR-WT and LHCSR3-Q mutant samples at pH 5 and pH 7.5 were retrieved from TRPL maps upon spectral integration. We attribute the slightly longer TRPL kinetics of the Q Mutant with respect to the LHCSR3-WT at pH 7.5 to a slightly higher concentration of loosely bound Chls which are not fully energetically connected to the other pigments within the protein.

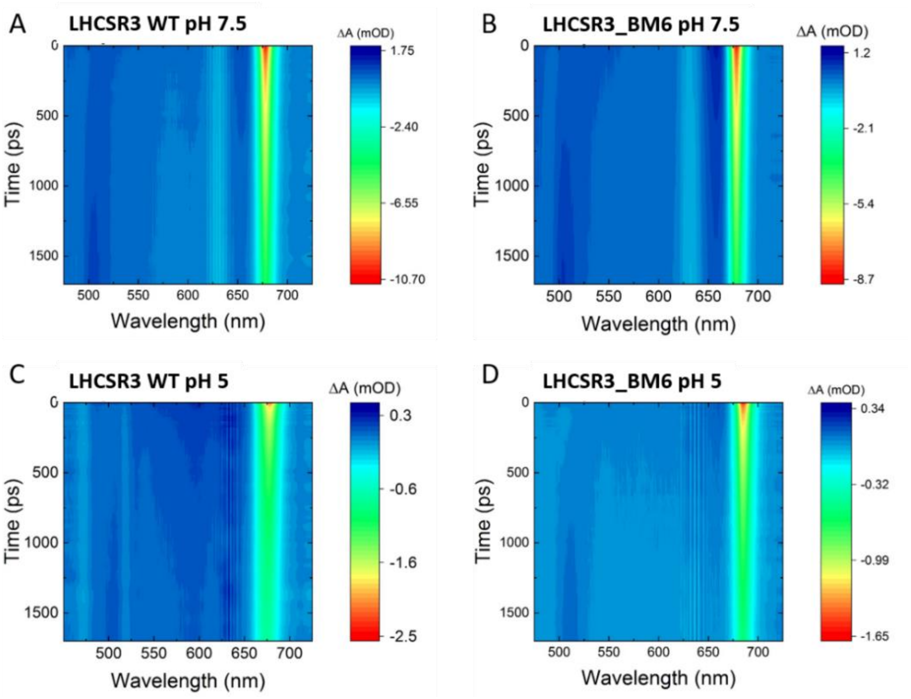

Figure S10. Visible TA maps. Visible TA maps (pump at 630 nm) of LHCSR3 WT pH 7.5 (A) and pH 5 (C); LHCSR3-BM6 pH 7.5 (B) and 5 (D).

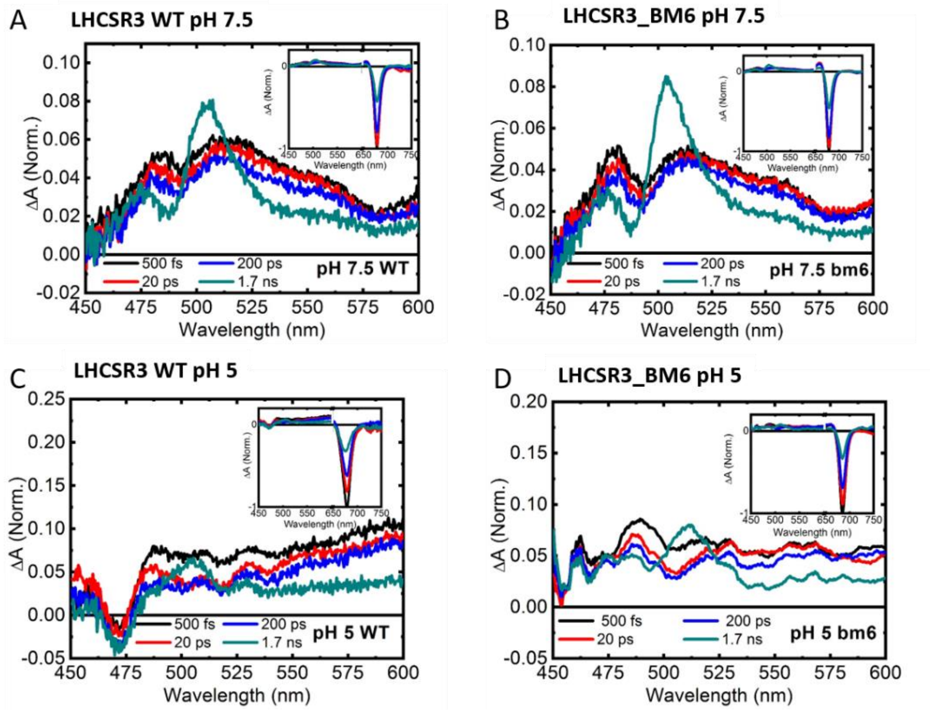

Figure S11. Visible TA spectra. Visible TA spectra of LHCSR3 WT pH 7.5 (A) and pH 5 (C); LHCSR3-BM6 pH 7.5 (B) and 5 (D). 500 fs, 20 ps, 200 ps and 1.7 ns after excitation are plotted. Whole TA spectra are plotted in each inset while the main graph represents a zoom into carotenoids region.

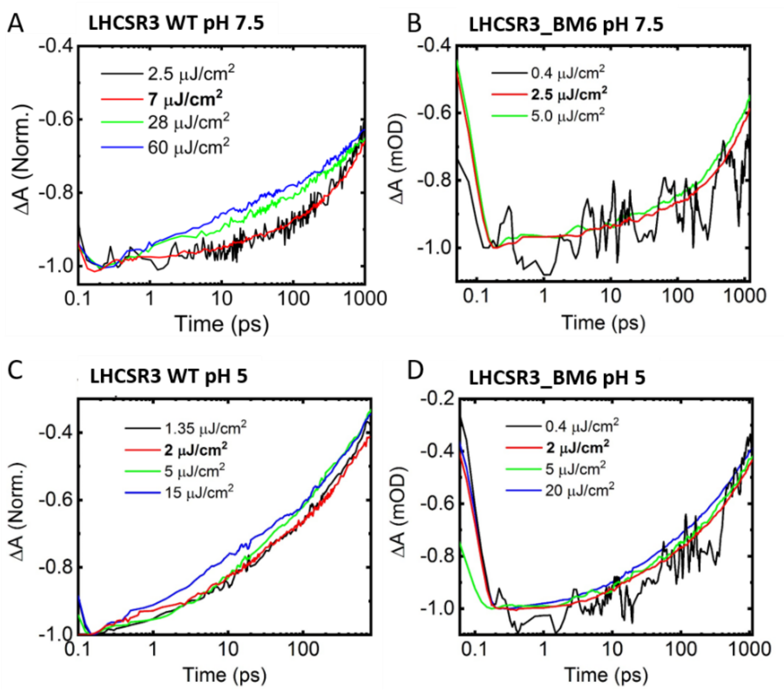

Figure S12. Fluence dependent TA dynamics. Fluence dependence for LHCSR3 WT and LHCSR3-BM6 at 680 nm. At the excitation fluence of 7 μJ/cm^2 for LHCSR3 WT pH 7.5 and 2 μJ/cm^2 for other samples used in the experiments, no singlet-singlet annihilation is detected.

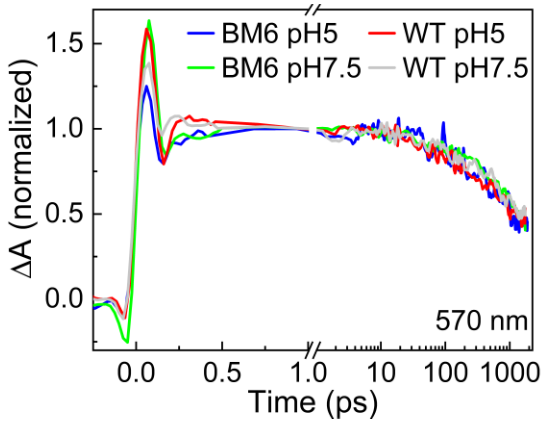

Figure S13. TA kinetic traces at 570 nm normalized at 1 ps.

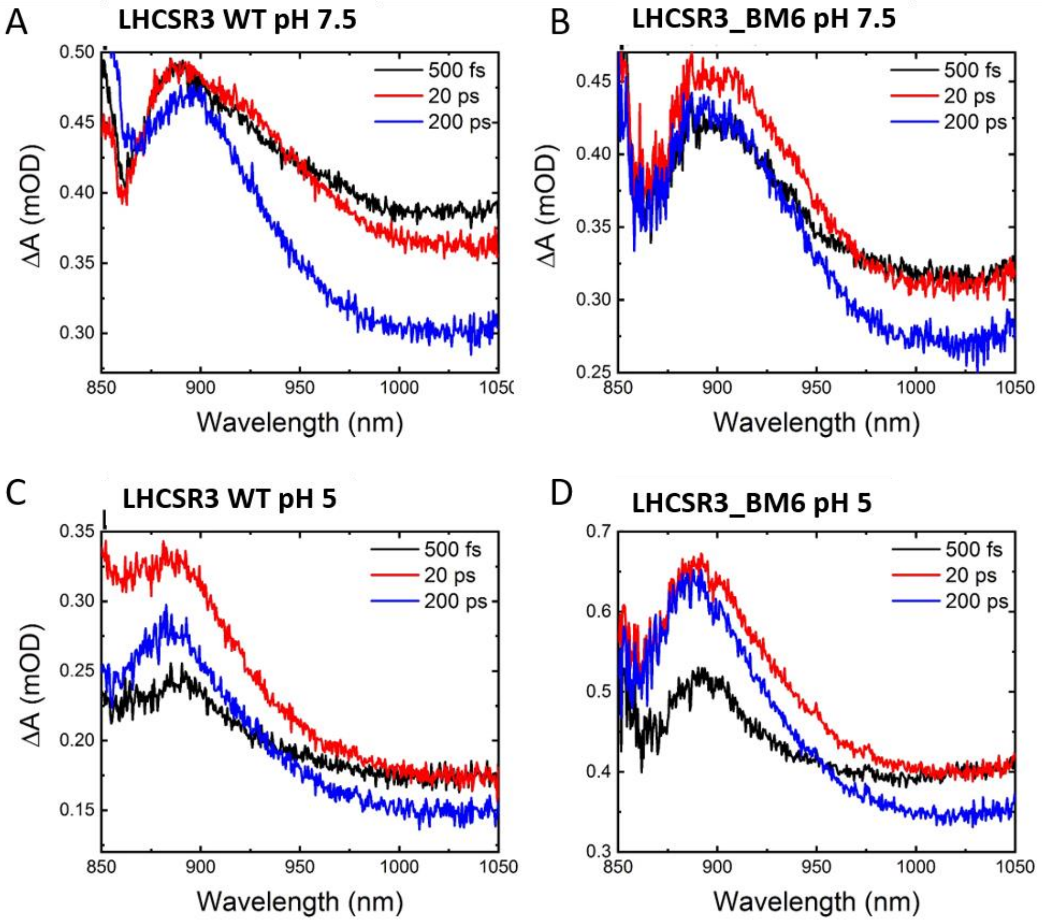

Figure S14. NIR TA spectra. NIR TA spectra of LHCSR3 WT pH 7.5 (A) and pH 5 (C); LHCSR3-BM6 pH 7.5 (B) and 5 (D). 500fs, 20ps and 200ps after excitation are plotted.

	Chl	Chl a	Chl b	Chl/Car	Car	violaxan-thin	anteraxan-thin	lutein	b-caro-tene
**LHCSR3 WT**	7	6.42 ± 0.37	0.57 ± 0.37	1.79	3.92	2.10 ± 0.23	0.07 ± 0.07	1.72 ± 0.13	0.04 ± 0.04
**LHCSR3-BM6**	7	6.20 ± 0.12	0.71 ± 0.12	2.06	3.4	1.94 ± 0.17	0.07 ± 0.02	1.37 ± 0.17	0.03 ± 0.02

Table S1. Pigment profiles. Pigment analysis of refolded LHCSR3 WT and LHCSR3-BM6 complexes. Chls: total number of chlorophylls; Cars: total number of carotenoids. Errors are reported as standard deviations (n=3).

	A_1 (%)	τ_1 (ps)	A_2 (%)	τ_2 (ps)	τ_{avg} (ps)
pH 7.5 LHCSR3 WT	8	185	92	3637	3369
pH 5 LHCSR3 WT	31	344	69	2279	1685
pH 7.5 LHCSR3-BM6	4	145	96	3620	3474
pH 5 LHCSR3-BM6	13	298	87	2952	2602

Table S2. Time constants. Time constants of LHCSR3 WT and LHCSR3-BM6 obtained by bi-exponential fits of TRPL maps in the whole spectral range.

References

(1) Ballottari, M.; Truong, T. B.; De Re, E.; Erickson, E.; Stella, G. R.; Fleming, G. R.; Bassi, R.; Niyogi, K. K. Identification of Ph-Sensing Sites in the Light Harvesting Complex Stress-Related 3 Protein Essential for Triggering Non-Photochemical Quenching in Chlamydomonas Reinhardtii. *J. Biol. Chem.* **2016**, *291*, 7334–7346.

(2) Pan, X.; Li, M.; Wan, T.; Wang, L.; Jia, C.; Hou, Z.; Zhao, X.; Zhang, J.; Chang, W. Structural Insights into Energy Regulation of Light-Harvesting Complex CP29 from Spinach. *Nat. Struct. Mol. Biol.* **2011**, *18*, 309–315.

(3) Roy, A.; Kucukural, A.; Zhang, Y. I-TASSER: A Unified Platform for Automated Protein Structure and Function Prediction. *Nat. Protoc.* **2010**, *5*, 725–738.

(4) Bonente, G.; Ballottari, M.; Truong, T. B.; Morosinotto, T.; Ahn, T. K.; Fleming, G. R.; Niyogi, K. K.; Bassi, R. Analysis of LhcSR3, a Protein Essential for Feedback de-Excitation in the Green Alga Chlamydomonas Reinhardtii. *PLoS Biol* **2011**, *9*, e1000577.

(5) Perozeni, F.; Cazzaniga, S.; Ballottari, M. In Vitro and in Vivo Investigation of Chlorophyll Binding Sites Involved in Non-photochemical Quenching in Chlamydomonas Reinhardtii. *Plant. Cell Environ.* **2019**, *42*, 2522–2535.

(6) Liguori, N.; Roy, L. M.; Opacic, M.; Durand, G.; Croce, R. Regulation of Light Harvesting in the Green Alga Chlamydomonas Reinhardtii: The c-Terminus of Lhcsr Is the Knob of a Dimmer Switch. *J. Am. Chem. Soc.* **2013**, *135*, 18339–18342.

(7) de la Cruz Valbuena, G.; Camargo, F. V. A.; Borrego-Varillas, R.; Perozeni, F.; D'Andrea, C.; Ballottari, M.; Cerullo, G. Molecular Mechanisms of Nonphotochemical Quenching in the LHCSR3 Protein of *Chlamydomonas Reinhardtii*. *J. Phys. Chem. Lett.* **2019**, *10*, 2500–2505.

(8) Lagarde, D.; Beuf, L.; Vermaas, W. Increased Production of Zeaxanthin and Other Pigments by Application of Genetic Engineering Techniques to Synechocystis Sp. Strain PCC 6803. *Appl. Environ. Microbiol.* **2000**, *66*, 64–72.
